# Glasgow Coma Scale in Brazilian Portuguese: foundation translation and cultural adaptation study

**DOI:** 10.31744/einstein_journal/2026AO2125

**Published:** 2026-09-23

**Authors:** Ana Camila de Castro Gandolfi, Patrícia Logullo, Moisés Cohen, Rachel Riera

**Affiliations:** 1 Department of Neurology and Neurosurgery Escola Paulista de Medicina Universidade Federal de São Paulo São Paulo SP Brazil Department of Neurology and Neurosurgery, Escola Paulista de Medicina, Universidade Federal de São Paulo, São Paulo, SP, Brazil.; 2 University of Oxford Bodleian Libraries Oxford United Kingdom University of Oxford, Bodleian Libraries, Oxford, United Kingdom.; 3 Orthopedics Department Escola Paulista de Medicina Universidade Federal de São Paulo São Paulo SP Brazil Orthopedics Department, Escola Paulista de Medicina, Universidade Federal de São Paulo, São Paulo, SP, Brazil.; 4 Department of Medicine Escola Paulista de Medicina Universidade Federal de São Paulo São Paulo SP Brazil Department of Medicine, Escola Paulista de Medicina, Universidade Federal de São Paulo, São Paulo, SP, Brazil.

**Keywords:** Brain concussion, Glasgow coma scale, Brain injuries, Brain injuries, traumatic

## Abstract

**Objective:**

To establish the foundation for the translation and culturally adaptation of the Glasgow Coma Scale into Brazilian Portuguese as part of a larger project translating and validating another tool, the SCAT-5, of which the Glasgow Coma Scale is a component. The Glasgow Coma Scale assesses the level of consciousness and neurological prognosis after traumatic brain injury. However, a systematic translation and cultural adaptation into Portuguese has never been performed.

**Methods:**

Three Brazilian individuals independently translated the original Glasgow Coma Scale from English to Portuguese. The Glasgow Coma Scale is divided into three separate modules: eye opening (scored between 1 and 4), verbal response (scored between 1 and 5), and best motor response (scored between 1 and 6). The total score is calculated by adding up the scores for each individual module, so it ranges between 3 and 15. Each sentence or expression within the original Glasgow Coma Scale questionnaire was evaluated individually. A multi-professional committee voted and discussed the best translation for each sentence or expression and combined them into a single version, which was back-translated into English by two separate native English professionals and compared with the original to resolve semantic discrepancies. The resulting combined and optimized version was tested twice in a fortnight and applied to 36 athletes who had suffered brain trauma, with cerebral concussion, and were treated in a sports-specialized ambulatory. This test was conducted to evaluate mainly the comprehension of the physician applying the translated questionnaire.

**Results:**

Patients were aged between 18 and 45 years, mostly rugby players (23/36), and female (23/36). There was no variability in the results between patients or between the same patient in both evaluations; all patients were consistently evaluated as having a Glasgow score of 15, since all patients were outpatients.

**Conclusion:**

This study laid the foundations for the translation and cultural adaptation of the Glasgow Coma Scale for Brazilian physicians. This translation may contribute to facilitating its use and standardizing the healthcare of patients with traumatic brain injury in Brazil. Further studies may be necessary to evaluate the use of the translated version in patients with lower levels of consciousness (i.e., lower Glasgow Coma Scale scores).

## Highlights

■ Glasgow Coma Scale translated and culturally adapted to Brazilian Portuguese.

■ Translated version tested on 36 patients at a cerebral concussion ambulatory in sports.

■ Translation accepted by the original Glasgow Coma Scale author and published at www.glasgowcomascale.org as the official Brazilian Portuguese version.

■ Further studies are necessary to evaluate the translated version in patients with a GCS score of <15.

## In Brief

Gandolfi et al. translated and culturally adapted the Glasgow Coma Scale into Brazilian Portuguese, thereby obtaining an adequate tool to assess the level of consciousness in the context of head trauma.

## Graphical abstract

**Figure:**
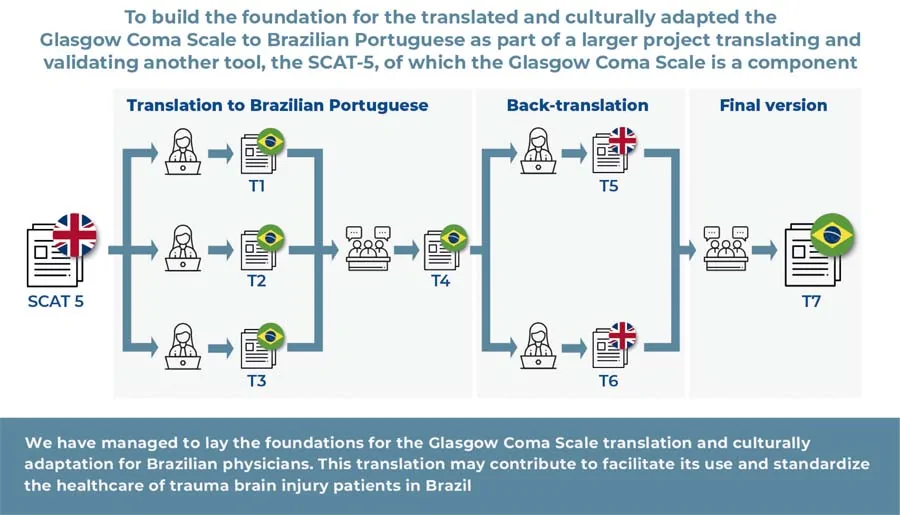

## INTRODUCTION

Traumatic brain injury (TBI) is an important public health issue worldwide because it is often preventable and leads to high rates of total or partial functional disability, often irreversible.^(1)^ Young adults, the population group with the highest levels of labor activity, are the most frequent victims of TBI, as it is related to accidents and violence. Men are more affected than women, with an estimated ratio of 3:1.^(2)^

In the United States, it is estimated that between 1.5 and 8 million people have TBI per year, and approximately 75%-90% are classified as having mild TBI.^(2)^ The mortality rate considering all TBIs is 3%, with more than 16% of the patients requiring hospitalization due to this trauma.^(3)^The initial assessment of the level of consciousness in patients with TBI is critical for decision-making in the emergency department and has prognostic value. In Brazil, as in most countries, the Glasgow Coma Scale (GCS)^(4)^ is used to make this assessment. However, to date, health professionals in Brazil have used the English version of the tool because of the unavailability of an adequate translated version.

Small variations in the score assigned to the patient on the GCS can change the patient’s overall classification (from mild to moderate TBI, for example)^(5-8)^ and the clinical approach. Therefore, it is important that there are no ambiguities in any sentence or expression within the scale, so that each question has only one possible answer for a given patient, regardless of the examiner.

The process of translating a diagnostic scale from the original (frequently in English) to the language in which it will be applied should not be a simple literal translation.^(9)^ It is essential that the translation is conducted by more than one individual and that a back-translation is carried out to resolve possible semantic and cultural differences. In addition, the method used for the translation process should avoid the inclusion of slang and/or idiomatic expressions that are untranslatable.^(9)^Applying the translated tool to potential patients helps to confirm whether, in the new language, that tool is suitable for evaluating what the users need to assess.

As the GCS has never been translated for use in Brazil, we report here its first translation, cultural adaptation, and testing in Brazilian Portuguese.^(10)^

## OBJECTIVE

Our objective was to lay the groundwork for obtaining an adequate tool in Brazilian Portuguese to assess the level of consciousness in the context of head trauma by translating and culturally adapting the Glasgow Coma Scale.

## METHODS

### Study design, setting, and ethics

This cross-sectional study was conducted in São Paulo, Brazil. The translated version was tested by applying it to athletes who were victims of brain trauma in a larger project aimed at translating and validating another tool, the SCAT-5, of which the GCS is a part. SCAT-5 is a tool used to assess cerebral concussion. This study was approved by the Independent Ethics Committee of the *Universidade Federal de São Paulo* (UNIFESP) (CAAE: 62592516.1.0000.5505; #1.865.130), and all patients signed an informed consent form (1624/2016).

The translation, cultural validation, and testing of the GCS into Brazilian Portuguese was performed between 2018 and 2020, after authorization by the main author and correspondent of the original GCS.

The GCS is divided into three separate modules: eye opening (scored between 1 and 4), verbal response (scored between 1 and 5), and best motor response (scored between 1 and 6). The total score is calculated by adding the scores for each individual module; therefore, it ranges between 3 and 15.

The overall methodologies proposed by Guilhemin et al.^(11,12)^ and Beaton et al.^(13)^ were used as detailed below (Figure 1).

**Figure 1:**
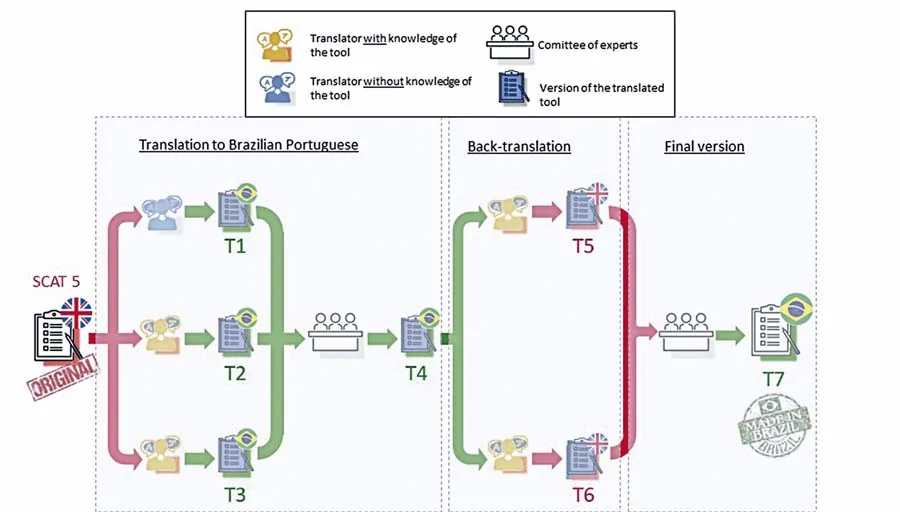
Translation and cultural adaptation process

Step 1: Translation - The initial GCS translation into Portuguese was performed independently by three Brazilians, two with prior knowledge of the tool and one without. The three versions (T1, T2, and T3) were generated. The three translators had no contact or discussion with each other.

Step 2: Expert committee review - A comparison between the three versions (T1, T2, and T3) was performed in a virtual environment (Google Forms), in which a committee of experts, self-declared fluent in English but native Portuguese speakers, was invited to select, in the electronic form, the best translation of each sentence/expression and resolve the linguistic and cultural discrepancies found between them, thereby generating T4, which is the result of the evaluation of three translations by a multidisciplinary committee, producing a single document translated into Portuguese that we call T4.

The professionals who voted did not know the identity of the translators. None of the eight committee members acted as translator.

Committee members were free to comment and suggest another translation alternative when none of the three options seemed best to them. They could also ask questions via email.

They had access to the full tool in the original English version when there was a need to consult the context to proceed with voting.

Any item in which more than 50% of the voters did not agree on the same option was submitted to a second round of voting only among the options that received the most votes.

Step 3: Back-translation into English - The T4 document approved by the committee was translated into English by two contracted translators, native speakers of English and with good knowledge of Portuguese, who had no knowledge of the study objectives. These two versions were named T5 and T6.

Step 4: Final version - Comparison of the three versions (T4 in Portuguese and T5 and T6 in English) was performed, with resolution of differences to improve clarity of the instructions. In addition to semantic equivalence (that is, the same meaning), aspects of idiomatic equivalence (there may be expressions that are impossible to translate, and in such cases new ones must be created) and the reality of Brazilians and their experiences (which may not be usual or normal in English-speaking environments) were evaluated. Thus, a final Portuguese version was produced based on T4 and corrected according to T5 and T6, which was used in the application for cultural validation. This version was called T7.

Step 5: Test application - Application of the T7 version to a group of 36 athletes invited to participate. The study included those who voluntarily or through medical referral sought the service with a possible neurological complaint or history of cerebral concussion. The participants underwent evaluation using the tool at the Cerebral Concussion Ambulatory in Sports at the Universidade Federal de São Paulo. The application was performed by physicians familiar with the tool.

Step 6: This is not a mandatory step in the health instrument translation process, but it is recommended that, for face validation of the tool, a statistical analysis be performed. In this case, the first and second assessments (test and retest) were compared.^(14,15)^

### Statistical analysis

The statistical analysis of the SCAT tool was performed by comparing patients’ responses in the first and second assessments (test and retest). However, as the participants were outpatients, the GCS assessment in Portuguese was identical in both evaluations (GCS=15, maximum score). This made it impossible to perform statistical analyses to assess agreement between the two assessments.

## RESULTS

### Translation

The initial translation from the original language (English) to the target language (Portuguese) generated T1, T2, and T3 versions; however, four sentences/expressions were translated equally by all translators and thus did not enter the Google Forms for voting. The committee had only two alternatives to choose from.

All Glasgow-related multiple-choice items reached a consensus (50% + 1) without the need for a second round of voting (as anticipated by the method).

Committee members were free to present suggestions and ask questions about the context of the test via email. Only one member suggested (and this was accepted) the inclusion of the terms “decortication” and “decerebration” in the GCS, as these terms are commonly used by Brazilian physicians who apply this scale. The suggested terms were added after slashes (translation according to voting with addition); that is, “extension in response to pain/decerebration” and “abnormal flexion in response to pain/decortication.”

### Test

The test was applied at a cerebral concussion ambulatory in sports. The inclusion criterion was a history of at least one concussion diagnosed in adulthood.

Of the 36 evaluated patients, there was a predominance of women (23:13), and most were rugby athletes (23 out of 36 patients). Their age ranged from 18 to 45 years, with a mean of 25.92 years (standard deviation of 5.26). The educational level of the participants ranged from high school completion to higher education, with 5% completing middle school, 64% completing high school, and 31% completing higher education.

All patients were evaluated using the tool twice, with a two-week period between each assessment. If the physicians had difficulty with a question repeatedly, the committee was consulted to make further modifications. The objective of this sequence of test applications was to ensure that at least 90% of the questions were adequately understood.

In the assessment using the GCS, there was no variability in results, with all patients evaluated with a GCS score of 15. For this reason, no statistical analysis was performed, since there was no variability between patients or between the same patient in both evaluations.

### Publication

The resulting translation of the Glasgow questionnaire was accepted by the main author and correspondent of the original GCS and published at www.glasgowcomascale.org as the official translation into Brazilian Portuguese (https://www.glasgowcomascale.org/downloads/GCS-Assessment-Aid-Brazilian.pdf).

## DISCUSSION

The coma assessment scale, better known as the GCS, was developed in Glasgow, Scotland, by Jennett and Teasdale in 1974 with the aim of being a paramedical scale for assessing the level of consciousness after cranioencephalic trauma.^(4)^ The scale was developed to standardize the assessment of the level of consciousness and to predict patient prognosis.^(5,6)^ In the emergency setting, the GCS is used not only in isolation but also as part of other scales, such as the Hunt-Hess scale, and as an auxiliary mechanism for decision-making in trauma patients.^(7)^

Small variations in the GCS can alter the medical management of a patient with a neurological disorder and modify the decision whether the patient should undergo surgery. In this context, it is critical that the GCS translation retains the same degree of objectivity as the original language.

The English language is quite widespread in Brazil, but it cannot, under any circumstances, be considered a second language. Using a tool in a language other than the patient’s or physician’s mother tongue can lead to inaccuracies in the assessment and even diagnostic errors. In addition, literal translations, without due technical care, could lead to loss of much-desired objectivity and practicality in the use of tools such as medical scales.

To ensure that an objective assessment can be made using the GCS in Brazil, it was fundamental to have a cultural validated translation available. For instance, the English term “confused” could be translated using its Portuguese cognate, “*confuso*.” However, “*confuso*” in Brazil has a broad meaning and could designate a person who is disoriented, sleepy, or speaking in a disconnected manner. For this reason, the committee decided to translate “confused” as “*desorientado*” (English: disoriented), as the word that best fits the criterion “not oriented but communicating coherently.”

The physicians applying the translated GCS were free to point out difficulties in understanding the text. The GCS questionnaire is used by the attending physician, who uses visual and verbal cues from the patient to fill in the correct score for each of the three GCS modules (explained above). In this process, the patient does not read the GCS questionnaire on their own, and the attending physician never reads from the questionnaire to the patient. Therefore, although the level of consciousness of the patient has little impact on the validation of the translation, it would be desirable to perform further studies, probably in emergency room situations, to completely validate the GCS in Brazil. The patients in this study were all outpatients; therefore, a preserved level of consciousness (i.e., GCS at 15) was already expected.

Nevertheless, this study provides a solid foundation for future studies that could assess the use of the translated scale in patients with a lower level of consciousness, which is likely to be found in hospital environments. Further studies should include patients with a wide range of GCS scores, such as those with moderate and severe TBI (GCS 13 or lower), and assess the degree of interobserver agreement, internal consistency, and other psychometric properties.

## CONCLUSION

The Glasgow Coma Scale was translated into Brazilian Portuguese and culturally adapted. Although further studies are necessary to evaluate the translated version in patients with a Glasgow Coma Scale score of less than 15, we have laid the groundwork for obtaining an adequate tool in Brazilian Portuguese to assess the level of consciousness in the context of head trauma.
